# Small Intestinal Fungal Overgrowth Mimicking a Surgical Abdomen and Presenting as Bowel Obstruction: A Case Report and Literature Review

**DOI:** 10.7759/cureus.105283

**Published:** 2026-03-15

**Authors:** Amaar Aamery, Hashim Ba Wazir, Mohammad Salem Amer, Elsadig Elmukashfi A Omer

**Affiliations:** 1 Department of General Surgery, Sultan Qaboos Hospital, Salalah, OMN; 2 Department of Medicine, Sultan Qaboos Hospital, Salalah, OMN; 3 Department of Radiology, Sultan Qaboos Hospital, Salalah, OMN; 4 Department of Surgery, Sultan Qaboos Hospital, Salalah, OMN

**Keywords:** antifungal therapy, chronic abdominal pain, gastrointestinal candidiasis, inflammatory stricture, intestinal mycosis, medical and surgical management, microbiome imbalance, small bowel obstruction, small-bowel resection, small intestinal fungal overgrowth

## Abstract

Small intestinal fungal overgrowth (SIFO) is defined by an abnormal proliferation of fungal organisms, most commonly *Candida* species, within the small intestine. Fungal infections, particularly candidiasis, are recognized causes of gastrointestinal (GI) symptoms, especially in patients with underlying conditions, such as malignancy or diabetes mellitus (DM), and in those exposed to immunosuppressive therapies, corticosteroids, or prolonged antibiotic use. SIFO is an underrecognized cause of GI symptoms, including bloating, diarrhea, and malabsorption. In rare cases, excessive fungal colonization can lead to mechanical bowel obstruction. This case report describes a patient with advanced retroviral infection who developed small bowel obstruction secondary to SIFO, underscoring the diagnostic challenges, therapeutic approaches, and the critical importance of early recognition and management.

## Introduction

Gut microbiota can be defined as the collection of all microorganisms in the human digestive system. There are 1014 CFU/mL of such microorganisms in the human body, including bacteria, viruses, and fungi [1]. The microbiota contributes to normal human physiology by facilitating digestion, supporting the development of the intestinal epithelium, maintaining intestinal barrier integrity, synthesizing essential vitamins, and providing protection against pathogenic organisms [1]. Any change in the composition or number of microorganisms, known as dysbiosis, disrupts the body’s homeostasis and can lead to the development of inflammatory bowel disease, irritable bowel syndrome, or metabolic diseases, such as diabetes mellitus (DM), obesity, and allergies [1]. Multiple types of disruptions to the gut microbiota have been identified: small intestinal bacterial overgrowth (SIBO), large intestinal bacterial overgrowth (LIBO), and small intestinal fungal overgrowth (SIFO) [1].

SIFO is characterized by the presence of excessive amounts of fungi in the small intestine associated with gastrointestinal (GI) symptoms. Fungal infections, particularly candidiasis, are well known to cause GI symptoms in patients with underlying diseases such as cancer, DM, and in those receiving steroids or antibiotics. Some studies have demonstrated that approximately 25.3% (38/150) of patients with unexplained GI symptoms had SIFO. Other studies suggested that SIBO and SIFO may coexist in up to 55% of patients [2].

Although commonly presenting with mild GI symptoms, such as bloating and diarrhea, severe cases of SIFO may result in mechanical obstruction of the bowel due to fungal biofilm formation, inflammation, and excessive fungal colonization. Understanding its pathophysiology and appropriate diagnostic approaches is essential for effective treatment.

Intestinal candidiasis rarely serves as the sole cause of bowel obstruction. Moreover, the absence of definitive diagnostic imaging criteria makes preoperative diagnosis uncommon, with the condition most often identified on histopathological examination of resected specimens [3]. Hence, SIFO should be included in the differential diagnosis for bowel obstruction in immunocompromised patients. As we mentioned earlier, although SIFO commonly presents with nonspecific GI symptoms, cases leading to bowel obstruction remain exceedingly rare and have been reported in only a handful of cases in the literature.

Here, we present a case of small bowel obstruction secondary to SIFO in an immunocompromised patient with advanced retroviral disease. Additionally, we provide a literature review exploring clinical presentations, diagnostic challenges, and current management strategies.

## Case presentation

A 22-year-old man was diagnosed with retroviral infection in August 2023 when he presented with weight loss (not quantified) and oral thrush. Baseline CD4 count was <40 cells/µL. He was started on anti-retroviral therapy (ART) consisting of efavirenz/tenofovir disoproxil fumarate/emtricitabine (EVF/TDF/FTC) in addition to a two-week course of fluconazole for oropharyngeal candidiasis and cotrimoxazole prophylaxis. Unfortunately, he could not tolerate ART due to neuropsychiatric side effects, and he stopped it by himself without reporting the side effects. He was lost to follow-up and presented again in February 2024 with approximately 12 kg of weight loss over three months, in addition to odynophagia. No significant oral thrush was noted. He was commenced on a different ART regimen consisting of ritonavir-boosted darunavir in addition to EVF/TDF/FTC, along with empiric fluconazole for suspected esophageal candidiasis. He came for a follow-up after two weeks, and odynophagia had resolved. Moreover, he was tolerating the ART regimen and had gained around 3 kg of weight.

In May 2024, he presented with recurrent attacks of post-prandial epigastric pain, distention, and vomiting. An abdominal X-ray revealed a dilated small bowel with air-fluid levels. He was admitted for evaluation. Esophagogastroduodenoscopy (EGD) showed features of *Candida* esophagitis and abundant fluid in the stomach, suggesting bowel obstruction. Colonoscopy was performed and was unremarkable.

**Figure 1:**
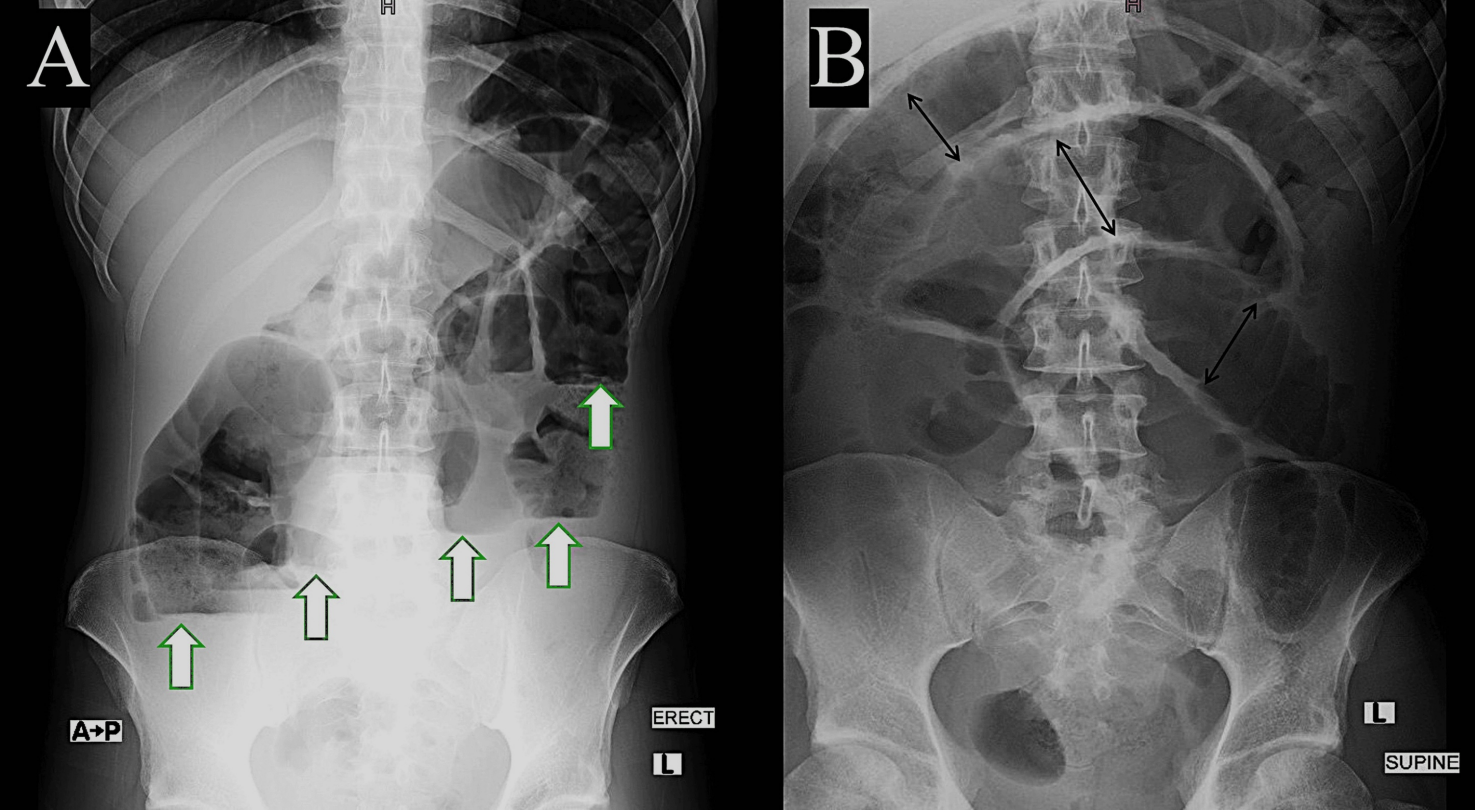
X-ray study of the abdomen, AP erect view (A), and AP supine view (B), showing multiple air-fluid levels (white arrows) and dilated small bowel loops (black arrows) AP: anterio-posterior.

**Figure 2:**
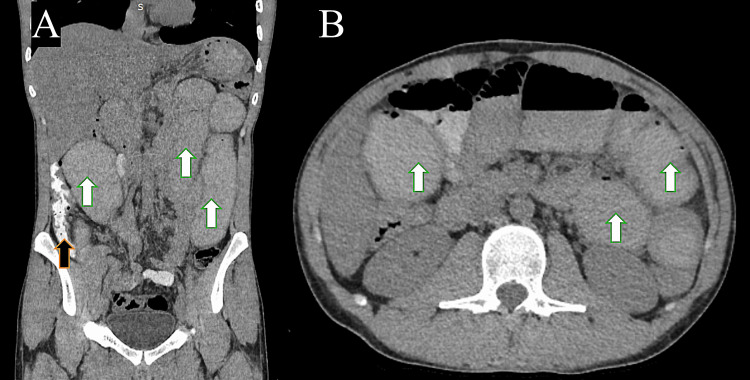
Plain computed tomography study of the abdomen with oral contrast, coronal (A) and axial cuts (B), showing dilated small bowel loops (white arrows). The transitional point is not well demarcated due to the limitations of the study. Oral contrast reaching the ascending colon (black arrow) suggests partial obstruction.

**Figure 3:**
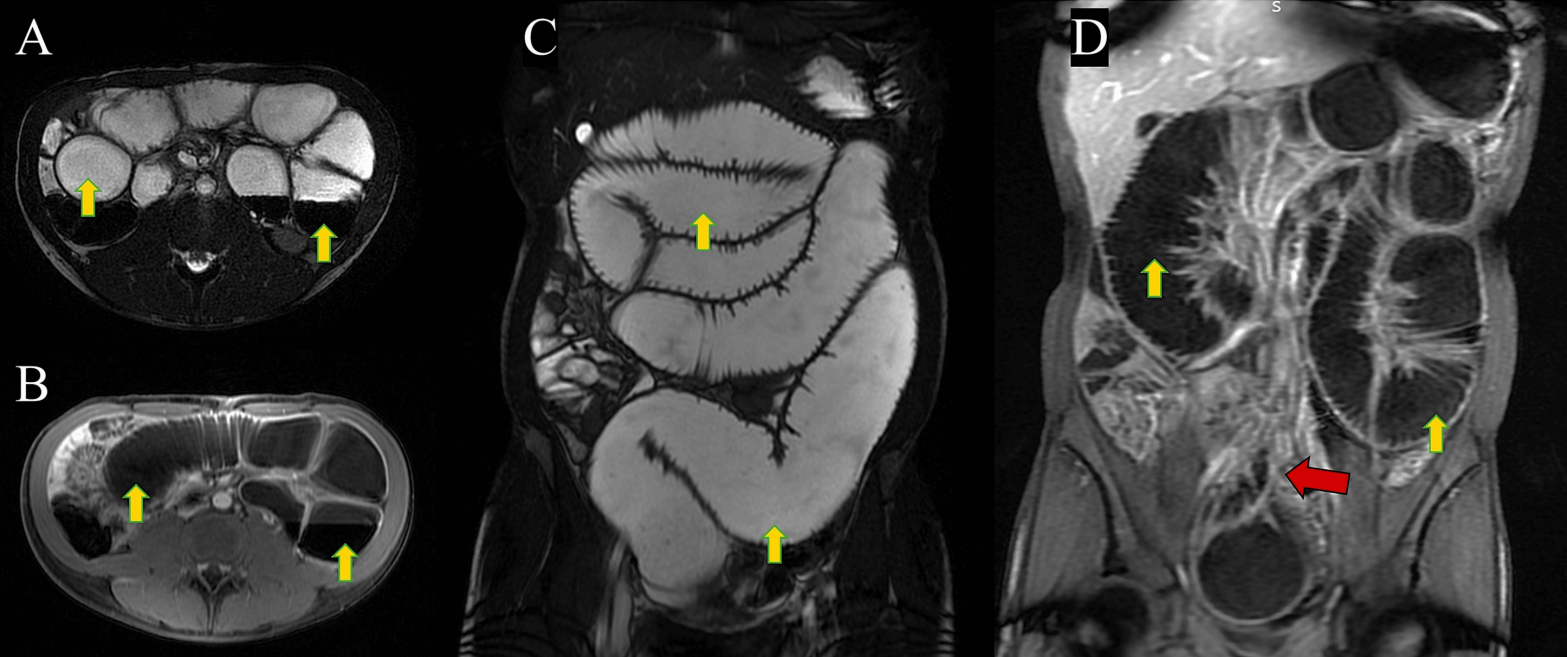
Magnetic resonance enterography study of the abdomen, axial FIESTA (A), axial LAVA +C (B), coronal FIESTA (C), and coronal LAVA +C (D) sequences, showing dilated small bowel loops (yellow arrows) with a transitional point at the right iliac fossa (red arrow). FIESTA: fast imaging employing steady-state acquisition; LAVA+C, liver acquisition with volume acquisition with contrast.

An abdominal X-ray revealed features of dilated bowel loops with air-fluid levels, indicating bowel obstruction as a diagnosis (Figure 1). Computerized tomography (CT) with oral contrast (without IV contrast due to mild pre-renal acute kidney injury) demonstrated small bowel dilatation without apparent cause and oral contrast in the ascending colon, suggesting only partial small bowel obstruction (Figure 2). Therefore, magnetic resonance enterography (MRE) was performed, which revealed the following: chronic partial small bowel obstruction at the level of the jejunum was suspected. Abrupt caliber narrowing of the ileal lumen near the right iliac fossa, zone of transition, was observed, leading to a collapsed cluster of ileal loops in the pelvic region. No specific inflammatory changes were observed in these segments. A fibrotic band was suspected (Figure 3). Based on these studies, the differential diagnoses considered were either a fibrotic band or an area of inflammatory bowel disease causing some localized stenosis.

During admission, the patient did not show signs of complete bowel obstruction; he was passing stool and flatus and tolerating oral intake. Therefore, no indication for urgent surgical intervention during admission was present when he was assessed by the general surgery team. Following improvement in symptoms, the patient was discharged, with planned outpatient follow-up to investigate the etiology of the radiological findings on CT and MRE.

**Figure 4:**
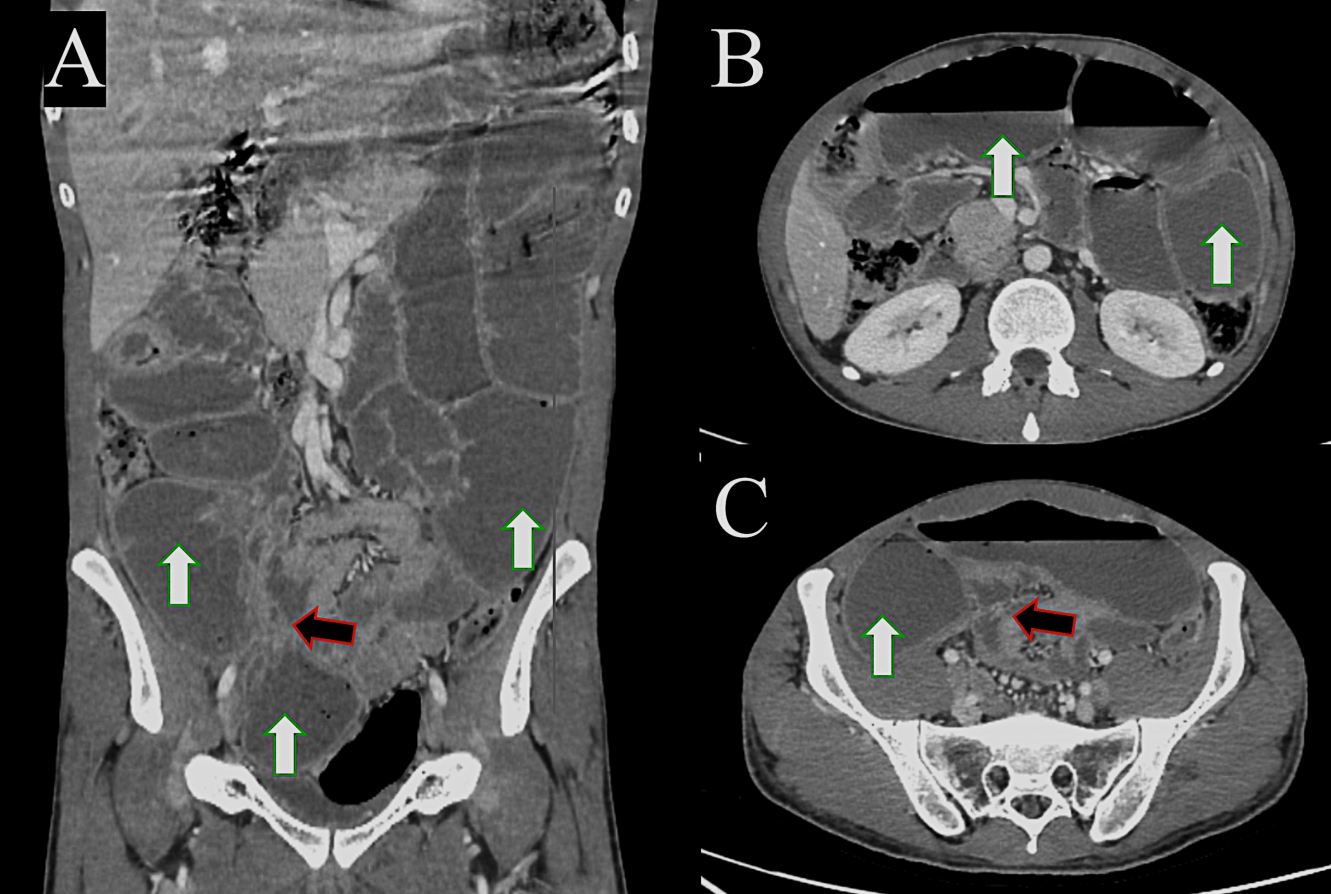
Contrast-enhanced computed tomography study of the abdomen, coronal (A), and axial cuts (B & C), showing dilated small bowel loops (white arrows) with a transitional point at the right iliac fossa (black arrow).

During outpatient follow-up, he continued to experience on-and-off symptoms. He was admitted in July 2024 with acute bowel obstruction after presenting with a three-day history of abdominal distension, pain, and vomiting. He was managed for bowel obstruction with nasogastric tube free drainage, intravenous fluid therapy, and urinary catheterization for input/output monitoring. An urgent CT abdomen/pelvis study revealed dilated and thickened proximal jejunal loops. A transit point was present with collapsed distal small bowel loops (Figure 4). Therefore, he was taken for an exploratory laparotomy as an emergency case.

Surgical intraoperative findings revealed a mid-small bowel stricture around 200 cm proximal to the ileocecal valve, causing small bowel obstruction with proximal dilated loops and distal collapsed ileum. No signs of bowel ischemia were present. Resection of the lesion (20 cm of small bowel) and primary end-to-end hand-sewn anastomosis (two layers) were performed.

The patient had a smooth postoperative course; he started oral feeding on the second postoperative day, which was well tolerated, and he was discharged on day 4 post-surgery.

Histopathology revealed chronic inflammation in the resected, narrowed small bowel segment with a stricture showing a fissuring ulcer, loss of the muscle layer, serosal fibrosis, and fungal colonization (morphologically resembling *Candida*). The resected bowel specimen was sent for acid-fast bacilli (AFB) staining, mycobacterial culture, and *Mycobacterium tuberculosis* complex polymerase chain reaction (MTBC-PCR). All three tests were negative. In addition, a fungal culture was performed, which revealed no fungal growth after two weeks of incubation. Based on the above results, a diagnosis of SIFO causing chronic small bowel inflammation and stricture was established. The patient was managed with fluconazole 300 mg once daily (OD) for four weeks.

On postoperative day 7, the patient developed a mild superficial surgical site infection and was evaluated in the surgical clinic. A small superficial collection was drained, and wound cultures yielded *Klebsiella pneumoniae*, which was susceptible to multiple antibiotics. The patient was treated with a short course of co-amoxiclav. He had an excellent recovery during the subsequent outpatient follow-up in the surgical clinic and was eventually discharged. He continued his periodic follow-ups in the Infectious Diseases (ID) clinic. No recurrence of small bowel obstruction was observed during the follow-ups; the patient was last reviewed in the ID clinic in December 2025. His CD4 count improved to >400 cells/µL, and HIV viral load remained suppressed.

## Discussion

SIFO is increasingly recognized as a contributor to chronic GI symptoms; however, its role in mechanical bowel obstruction remains poorly understood. The pathophysiology of SIFO-related obstruction likely involves fungal biofilm formation, inflammation, and an element of altered intestinal motility.

Fungal overgrowth in the gut has been described in patients with immunocompromised status, such as those with HIV infection, DM, malignancy, and others. The most frequently isolated fungal organism in these cases is *Candida*, with *Candida albicans* identified as the predominant species [2]. Although it is commonly found as a commensal, *Candida *enteritis*,* causing acute intestinal obstruction, has been very rarely reported [2].

In a study of 890 necropsies, Prescott et al. identified mycotic infections in 7.2% of cases. Among these, 26 involved the GI tract, with 12 occurring in the upper gut, seven in the mid-to-lower gut, and seven presenting as multi-site infections across both regions [4].

Clinical presentation can vary from mild/moderate symptoms, such as abdominal pain, abdominal distension, gas, flatulence, and diarrhea, which are the most common symptoms reported by patients with SIFO. Malabsorption may occur, resulting in fatigue and nutritional deficiencies, whereas severe cases can manifest with features of bowel obstruction, including severe abdominal pain, vomiting, constipation, and abdominal distension. The etiology of small bowel obstruction can be explained by a thick fungal biofilm in the bowel lumen, causing narrowing, combined with the inflammatory response associated with the infection. Intestinal candidiasis has been linked to a myriad of systemic symptoms that overlap with SIBO, such as migraine, fatigue, depression, and bloating [5]. In our patient, the only systemic symptom was fatigue, which was attributed to the malnutrition.

Preoperative diagnosis requires a high index of suspicion, particularly in immunocompromised patients presenting with bowel obstruction. CT can show dilated bowel loops and bowel wall thickening in the involved segment of the intestine. A definitive diagnosis typically requires histopathological examination of tissue samples obtained during surgical intervention [6]. Currently, preoperative diagnosis of SIFO can be achieved through duodenal aspiration with fungal culture, which remains the only available diagnostic modality apart from surgical excisional biopsy [2].

Treating SIFO focuses on reducing fungal burden, addressing predisposing factors, and preventing recurrence. Factors such as the severity of candidiasis, intolerance to antifungal treatment, any underlying comorbidities, and the patient’s immunity and clinical stability must be considered [1]. Antifungal therapy with fluconazole may be effective in small intestinal fungal overgrowth. The optimal dosing regimen and treatment duration in SIFO cases have not yet been clearly established; however, a treatment course of 10-14 days has been suggested [2]. In patients with *Candida* infection of other parts of the GI, such as the esophagus, a 14- to 21-day course is usually advised. However, this can be extended to one month in patients who are immunocompromised or have recurrent or refractory disease, as in our patient. Dietary modification, including a protein-rich, carbohydrate-deficient diet, has demonstrated some therapeutic benefit as part of multimodal treatment, particularly in persistent or recurrent cases.

In cases similar to our patient, who presented with chronic bowel obstruction, a presentation rarely described in the literature, no specific guidelines addressing whether medical therapy alone can resolve small bowel lesions and alleviate obstructive symptoms exist. In our case, the delay in diagnosis due to the unavailability of a specific diagnostic modality preoperatively might have contributed to small bowel obstruction, caused by worsening fungal biofilm and associated bowel inflammation. Hence, in cases where patients exhibit persistent symptoms despite empirical therapy or when a definitive diagnosis cannot be established, surgical intervention represents the sole viable approach. Surgery provides both diagnostic confirmation of bowel candidiasis and facilitates the initiation of targeted antifungal therapy, as well as therapeutic relief of obstructive manifestations.

## Conclusions

SIFO is an underrecognized yet clinically significant condition that, in rare cases, can cause bowel obstruction. Management of this condition presents a clinical dilemma due to associated patient comorbidities and the challenges inherent in establishing a definitive diagnosis preoperatively. Including SIFO on the list of differential diagnoses for bowel obstruction in immunocompromised patients could lead to earlier surgical intervention, earlier histopathological diagnosis, and ultimately better patient outcomes. Increased awareness and improved diagnostic strategies are essential for effective management. Future research should focus on non-invasive diagnostic tools and the role of microbiome-targeted therapies in enhancing patient outcomes.
